# HSPB1, HSPB6, HSPB7 and HSPB8 Protect against RhoA GTPase-Induced Remodeling in Tachypaced Atrial Myocytes

**DOI:** 10.1371/journal.pone.0020395

**Published:** 2011-06-24

**Authors:** Lei Ke, Roelien A. M. Meijering, Femke Hoogstra-Berends, Katarina Mackovicova, Michel J. Vos, Isabelle C. Van Gelder, Robert H. Henning, Harm H. Kampinga, Bianca J. J. M. Brundel

**Affiliations:** 1 Department of Radiation and Stress Cell Biology, University Institute for Drug Exploration (GUIDE), University of Groningen, University Medical Center Groningen, Groningen, The Netherlands; 2 Department of Clinical Pharmacology, GUIDE, University of Groningen, University Medical Center Groningen, Groningen, The Netherlands; 3 Nyken BV, Groningen, The Netherlands; 4 Department of Cardiology, GUIDE, University of Groningen, University Medical Center Groningen, and the Interuniversity Cardiology Institute Netherlands, Utrecht, The Netherlands; University of Illinois at Urbana-Champaign, United States of America

## Abstract

**Background:**

We previously demonstrated the small heat shock protein, HSPB1, to prevent tachycardia remodeling in *in vitro* and *in vivo* models for Atrial Fibrillation (AF). To gain insight into its mechanism of action, we examined the protective effect of all 10 members of the HSPB family on tachycardia remodeling. Furthermore, modulating effects of HSPB on RhoA GTPase activity and F-actin stress fiber formation were examined, as this pathway was found of prime importance in tachycardia remodeling events and the initiation of AF.

**Methods and Results:**

Tachypacing (4 Hz) of HL-1 atrial myocytes significantly and progressively reduced the amplitude of Ca^2+^ transients (CaT). In addition to HSPB1, also overexpression of HSPB6, HSPB7 and HSPB8 protected against tachypacing-induced CaT reduction. The protective effect was independent of HSPB1. Moreover, tachypacing induced RhoA GTPase activity and caused F-actin stress fiber formation. The ROCK inhibitor Y27632 significantly prevented tachypacing-induced F-actin formation and CaT reductions, showing that RhoA activation is required for remodeling. Although all protective HSPB members prevented the formation of F-actin stress fibers, their mode of action differs. Whilst HSPB1, HSPB6 and HSPB7 acted via direct prevention of F-actin formation, HSPB8-protection was mediated via inhibition of RhoA GTPase activity.

**Conclusion:**

Overexpression of HSPB1, as well as HSPB6, HSPB7 and HSPB8 independently protect against tachycardia remodeling by attenuation of the RhoA GTPase pathway at different levels. The cardioprotective role for multiple HSPB members indicate a possible therapeutic benefit of compounds able to boost the expression of single or multiple members of the HSPB family.

## Introduction

Atrial Fibrillation (AF) is the most common sustained and progressive clinical tachycardia in the population and it significantly contributes to cardiovascular morbidity and mortality [1]. AF is characterized by specific changes in electrical, structural and conctractile function of the atrial myocytes, commonly denoted as ‘remodeling’. Tachycardia remodeling underlies contractile dysfunction and the progressive and intractable nature of AF. Therefore, remodeling is believed to have important therapeutic implications, and there is great interest in developing anti-remodeling therapies directed at the targets underlying remodeling [1].

We recently identified one specific member of the heat shock protein (HSP) family, HSPB1, to protect against AF-induced remodeling [2]; [3]. HSPs are molecular chaperones and prevent the accumulation of the misfolded or unfolded proteins in the cells [4]. HSPB1 is one member of the small heat shock protein (sHSP or HSPB in mammals) family, which comprises a total of at least ten members [5]; [6]. A characteristic of most HSPBs is their ability to interact with components of the actin cytoskeleton, and this binding protects against cytoskeletal injury during stress, resulting in conservation of the cell function [7]. In addition, HSPBs collectively share important features, including (1) a conserved α-crystallin domain, (2) ability to form large oligomers *in vitro* and (3) increased expression upon exposure to various stresses including heat stress [8]. Nevertheless, the precise mode of action of HSPB1 to protect from tachycardia remodeling remains elusive and it is unknown whether this is shared between other members of the HSPB family. Therefore, we examined if, in addition to HSPB1, also other HSPB members protect against atrial tachycardia remodeling. Hereto, we utilized tachypaced HL-1 myocytes, an *in vitro* atrial cell line model for tachycardia remodeling [3]; [9]. In addition to HSPB1, we identified HSPB6, HSPB7 and HSPB8 to protect against tachypacing-induced calcium transient reduction. Because of the known protective actions of HSPBs on actin cytoskeleton, we next examined their effect on tachypacing-induced RhoA GTPase pathway, including RhoA GTPase activity and related F-actin stress fiber formation. Although all protective HSPB members reduced the formation of F-actin stress fibers, their mode of action differs. HSPB1, HSPB6 and HSPB7 were found to directly prevent F-actin stress fiber formation, whereas HSPB8-protection was mediated via inhibition of upstream RhoA GTPase activity.

## Materials and Methods

### 2.1. HL-1 atrial myocyte culture, transfections and constructs

HL-1 atrial myocytes, derived from adult mouse atria, were obtained from Dr. William Claycomb as described before [3]. The myocytes were maintained in Complete Claycomb Medium (JRH, UK) supplemented with 100 µM norepinephrine (Sigma, The Netherlands), 0.3 mM L-ascorbic acid (Sigma), 4 mM L-glutamine (Gibco, The Netherlands) and 10% FBS (Life Technologies, Gaithersburg, MD). They were cultured on coverslips coated with 12.5 µg/ml fibronectin (Sigma) and 0.02% gelatin (Sigma), in a 5% CO_2_ atmosphere at 37°C.

To study the influence of HSPBs on Ca^2+^ transient changes, HL-1 myocytes were transiently (co-)transfected, by the use of Lipofectamin (Life Technologies, The Netherlands), with the plasmid CD8 cDNA encoding CD8 antigen and/or pCDNA5/FRT/TO-HSPBX (X indicating 1-10) encoding human HSPB members. Positive myocytes were selected by anti-CD8 Dynabeads (Dynal). To check overexpression of HSPBX proteins in HL-1 myocytes, myocytes were transiently transfected with the fusion proteins V5-HSPBX. For all other experiments HSPBX wildtype constructs were used.

### 2.2. Tachypacing of HL-1 myocytes

HL-1 myocytes were subjected to tachypacing as described before [2]; [3]; [9]. In short, the spontaneous rate of HL-1 myocytes is ∼1 Hz. HL-1 myocytes were subjected to normal electrical field stimulation (1 Hz) for at least 30 min before tachypacing via the C-Pace100™-Culture Pacer (IonOptix Corporation, The Netherlands). Tachypacing was performed at 4 Hz with 20-ms pulses for 8 hours to induce CaT reduction and 1 Hz pacing was used as a control.

### 2.3. Protein-extraction and Western blot analysis

Western-blot analysis was performed as described previously [2]; [3]. Equal amount of protein in SDS-PAGE sample buffer was sonicated before separation on 10% PAA-SDS gels. After transfer to nitrocellulose membranes (Stratagene, The Netherlands), membranes were incubated with primary antibodies against HSPB1 (SPA801, StressGen USA), V5 tag (Invitrogen, The Netherlands) or GAPDH (Affinity Reagents, The Netherlands). Horseradish peroxidase-conjugated anti-mouse or anti-rabbit (Santa-Cruz Biotechnology, The Netherlands) was used as secondary antibody. Signals were detected by the ECL-detection method (Amersham, The Netherlands) and quantified by densitometry.

### 2.4. Live imaging and measurement of CaT

To measure CaT, 2 µM of the Ca^2+^-sensitive Fluo-4-AM dye (Invitrogen, The Netherlands) was loaded into HL-1 myocytes by 45 min incubation, followed by 3 times washing with DMEM solution. Ca^2+^ loaded myocytes were excited by 488 nm and light emitted at 500–550 nm and visually recorded with a 40x-objective, using a Solamere-Nipkow-Confocal-Live-Cell-Imaging system (based on a Leica DM IRE2 Inverted microscope). The live recording of CaT in HL-1 myocytes was performed at 1 Hz of stimulation in a temperature (37°C) controlled system. By use of the software ImageJ (National Institutes of Health, USA), the absolute value of fluorescent signals in live myocytes were recorded and analyzed. To compare the fluorescent signals between experiments, the following calibration was utilized: F_cal_ = F/F0, in which (F) is fluorescent dye at any given time and (F0) is fluorescent signal at rest [10]. Mean values from each experimental condition were based on 7 consecutive CaT in at least 50 myocytes.

### 2.5. Immunofluorescent staining and confocal analysis

Twenty-four hours after transient transfection of HSPB1, HSPB5, HSPB6, HSPB7 and HSPB8, HL-1 myocytes were subjected to (tachy)pacing. Afterwards, the myocytes were fixed with 3.7% formaldehyde for 15 minutes, washed three times with Phosphate-Buffered Saline (PBS), permeabilized with 0.2% Triton-X100 and blocked with 0.1 glycine (10 minutes at room temperature) and 5% BSA (30 minutes at room temperature). Antibodies against HSPB6, HSPB7 and HSPB8 (all Abcam, The Netherlands), and HSPB1 and HSPB5 (StressGen, USA) were used as primary antibody. Fluorescein labeled isothiocyanate (FITC) anti-mouse or anti-rabbit (Jackson ImmunoResearch, The Netherlands) were used as secondary antibodies. To visualize F-actin, rhodamine phalloidin (Invitrogen, The Netherlands) was diluted with PBS at 1∶40, followed by incubation for 20 minutes at room temperature and washed three times with PBS. Images of FITC and rhodamine fluorescence were obtained using the Leica confocal laser scanning microscope (Leica SP2 AOBS) with 63X/1.4 oil lens. The captured images were processed using Leica Confocal Software and Adobe Photoshop. For determination of the amount of F-actin stress fibers, the intensity of fluorescence was analyzed by ImageJ in 5 independently taken fields. HL-1 myocytes were treated 4 hours prior and during (tachy)pacing with the ROCK inhibitor Y27632 (10 µM, Sigma, The Netherlands) to prevent F-actin stress fiber formation. For determination of the amount of colocalization of HSPB with stress fibers, ImagePro software was used. The amount of colocalization was determined as the ratio of total red signal (F-actin) divided by yellow signal (colocalization HSPB with F-actin). Between 400 and 500 myocytes were quantified per condition.

### 2.6. Short interfering RNA of HSPB1 in combination with over-expression of HSPB6, HSPB7 or HSPB8

Downregulation of endogenous HSPB1 was performed as described previously [3]. HL-1 myocytes were transiently transfected with HSPB1 siRNA or mock constructs for 5 days. Furthermore, 24 hours before tachypacing, cells were co-transfected with HSPB6, HSPB7 or HSPB8 construct. After 8-hours (tachy)pacing, CaT were measured and analyzed.

### 2.7. RhoA GTPase activity measurement with G-LISA

For the quantitative analysis of active RhoA GTP levels, G-LISA RhoA Activation Assay (Cytoskeleton, USA) was performed according to the manufacturer protocol. Briefly, 48 hours after the transfection of HL-1 myocytes, myocytes were subjected to (tachy)pacing for 6 hours or directly lysed in lysis buffer and cells were harvested. After measurement of the protein concentration with the use of Precision Red (supplied), equal amounts of lysates were incubated in RhoA GTP affinity plates. The amount of bound RhoA GTP was detected by using primary anti-RhoA antibody (1∶250, supplied) and secondary HRP-labeled antibody (1∶62.5, supplied). Colorimetric detection at 490 nm was performed immediately (BioRad, The Netherlands).

### 2.8. Actin (de-)polymerization-assay

To determine the direct effect of HSPBs on actin polymerization and depolymerization an actin polymerization biochem kit (Cytoskeleton, USA) was used. Twenty-four hours after transient transfection of HL-1 myocytes with HSPB1, HSPB5, HSPB6, HSPB7 or HSPB8, myocytes were lysed in a mild lysis buffer according to the manufacturer protocol. As a control, recombinant human HSPB1 (Stressgen, USA) was dissolved in lysis buffer. Base-line fluorescence of pyrene conjugated actin was measured (Ex. 350 nm; Em. 405 nm) for three minutes, after which cell lysates and recombinant HSPB1 were added to measure effect on (de-)polymerization. Fluorescence was assayed every 60 s for twenty minutes. Maximum actin polymerization was determined by adding polymerization buffer.

### 2.9. Statistical analysis

Results are expressed as mean ± SEM. All CaT measurements were performed in at least triple series. Mean values from each experimental condition were based on 7 consecutive CaT in at least 50 myocytes. ANOVA was used for multiple-group comparisons. All p-values were two-sided. P<0.05 was considered statistically significant. SPSS version 16.0 was used for statistical evaluation.

## Results

### 3.1. Effect of overexpression of the ten different HSPB members on tachypacing-induced remodeling in HL-1 myocytes

**Figure 1:**
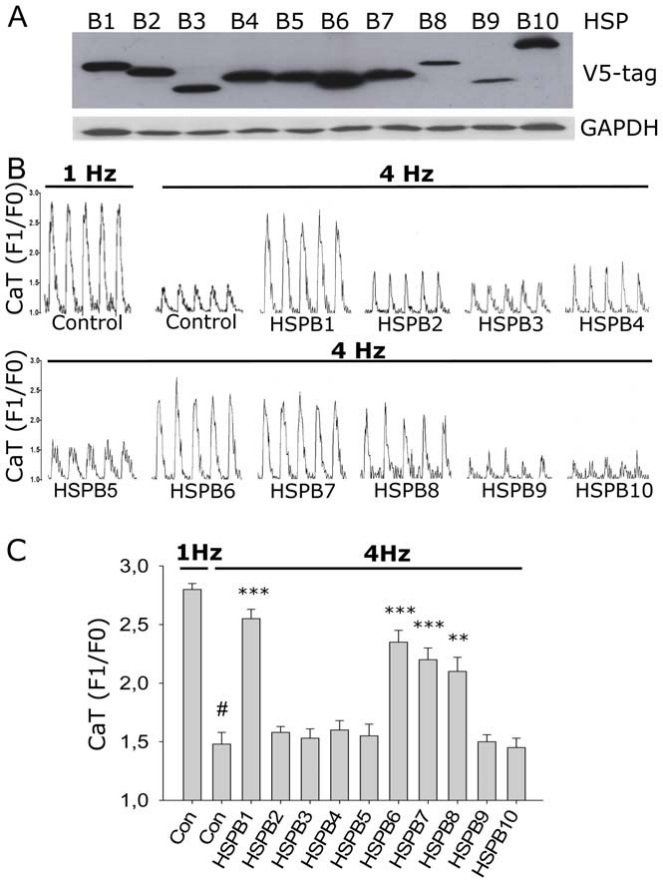
HSPB1, HSPB6, HSPB7 and HSPB8 overexpression prevents against tachypacing-induced CaT reductions in HL-1 myocytes. (A) Representative Western blot showing overexpression of HSPB1-10 in transiently transfected HL-1 myocytes. (B) Original recordings of CaT in 1 myocyte each from groups indicated. (C) Mean CaT data of HSPB1-10 overexpressing myocytes tachypaced (4 Hz) or normal paced cells (1 Hz). **P<0.01, ***P<0.001 vs control tachypaced (4 Hz). # P<0.001 vs control normal paced (1 Hz).

In humans, the HSPB family comprises a group of 10 members with monomeric molecular weight varying between 16 to 28 kDa [5]; [6]; [8]. Induction of HSPB1 has been shown previously to protect against atrial tachypacing-induced remodeling, including CaT reduction [3]. To study the effect of individual HSPB members, HL-1 myocytes were transfected with V5 tagged constructs for each member. All members were successfully overexpressed, albeit HSPB8 and HSPB9 at a lower level (Figure 1A). As a control group, HL-1 myocytes were transfected with an empty vector. None of the overexpressed HSPB members changed CaT in control myocytes paced at 1 Hz (data not shown). As observed before [3], tachypacing at 4 Hz of HL-1 myocytes resulted in a significant and progressive reduction in CaT (Figure S1), which was attenuated by HSPB1 (Figure 1B,C Movie S1, Movie S2, Movie S3). In addition, overexpression of HSPB6, HSPB7 and HSPB8 also protected against tachypacing-induced CaT depression, whereas the other members were ineffective (Figure 1B,C and Movie S1, Movie S2, Movie S3, Movie S4, Movie S5, Movie S6, Movie S7, Movie S8, Movie S9, Movie S10, Movie S11, Movie S12). These results indicate that in addition to HSPB1 also HSPB6, HSPB7 and HSPB8 protect against tachypacing-induced CaT reduction.

### 3.2. HSPB6, HSPB7 and HSPB8 protection against tachypacing-induced CaT reduction is independent of endogenous HSPB1 expression

**Figure 2:**
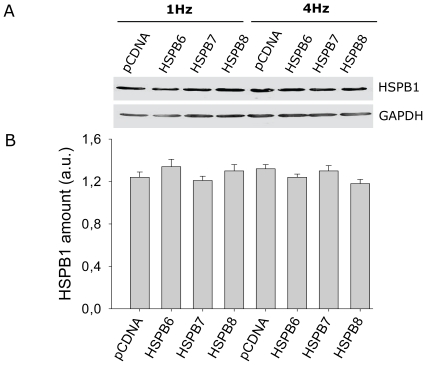
Overexpression of HSPB6, HSPB7 or HSPB8 do not result in changes in endogenous HSPB1 levels. (A) Representative Western blot showing that the endogenous HSPB1 levels in transfected HSPB6, HSPB7 and HSPB8 overexpressing HL-1 myocytes are not changed in normal paced myocytes (1 Hz) or tachypaced myocytes (4 Hz). (B) Corresponding mean data (n = 3 experiments/group).

**Figure 3:**
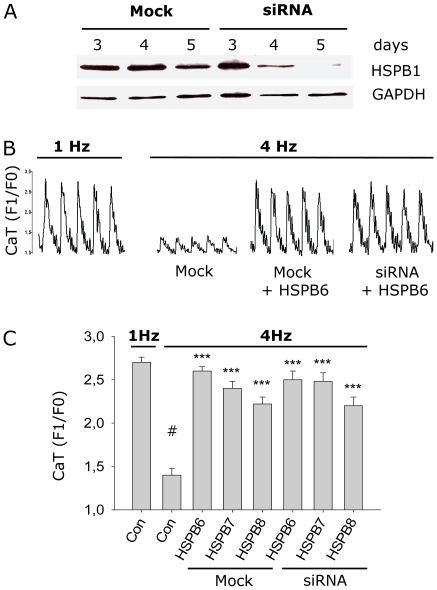
HSPB6, HSPB7, HSPB8 protective effect is independent of HSPB1. (A) Western blot showing efficient siRNA-induced HSPB1 knockdown in HL-1 mycoytes. (B) Recordings of CaT for mock and siRNA construct. HL-1 myocytes were transfected with mock or siRNA construct for 5 days before study. One day before tachypacing, the myocytes were transfected with HSPB6, HSPB7 or HSPB8 and subjected to normal pacing (1 Hz) or tachypacing (4 Hz). (C) Mean CaT data. The cardioprotective effect of HSPB6, HSPB7 or HSPB8 was not blocked by HSPB1 suppression. ***P<0.001 vs control tachypaced (4 Hz). # *P*<0.001 vs control normal paced (1 Hz).

HSPB members are known for their ability to form hetero-oligomeric complexes [11]; [12] and given the fact that HSPB1 is constitutively expressed in HL-1 myocytes, the possibility existed that the protective effect of HSPB6, HSPB7 or HSPB8 on tachypacing-induced CaT reduction was related to (indirect) effects via (oligomerization with) HSPB1. Also, ectopic HSPB expression may induce a stress response in cells leading to the up-regulation of endogenous HSPB1. To exclude these possibilities, it was first determined whether overexpression of HSPB6, HSPB7 or HSPB8 increased expression of endogenous HSPB1 levels. As shown in Figure 2, endogenous HSPB1 levels were similar after normal pacing (1 Hz) and tachypacing (4 Hz), irrespective of HSPB6, HSPB7 or HSPB8 overexpression. Secondly, the endogenous HSPB1 level was suppressed by short hairpin RNAs (Figure 3A). In HSPB1 depleted myocytes, HSPB6, HSPB7 or HSPB8 overexpression could still protect against tachypacing-induced CaT reduction (Figure 3B,C, Movie S13, Movie S14, Movie S15, Movie S16, Movie S17, Movie S18). In summary, these results suggest that the protective effects of HSPB6, HSPB7 and HSPB8 against tachypacing-induced CaT reduction are independent of HSPB1.

### 3.3. HSPB6, HSPB7 and HSPB8 reduce the amount of F-actin stress fibers after tachypacing in HL-1 myocytes

**Figure 4:**
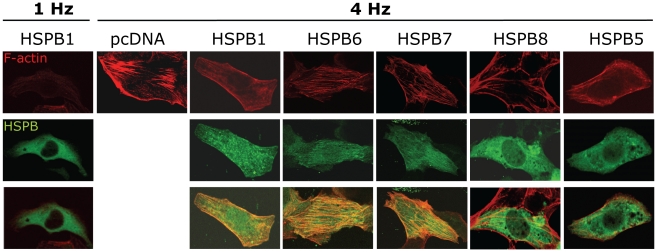
HSPB1, HSPB6 and HSPB7 coocalize with tachypacing-induced F-actin stress fibers in HL-1 myocytes. Immunofluorescent staining of F-actin stress fibers (red) and HSPB positive myocytes (green), in tachypaced HL-1 myocytes (4 Hz). A normal paced (1 Hz) HSPB1 transfected myocyte was shown as a representative control example.

**Figure 5:**
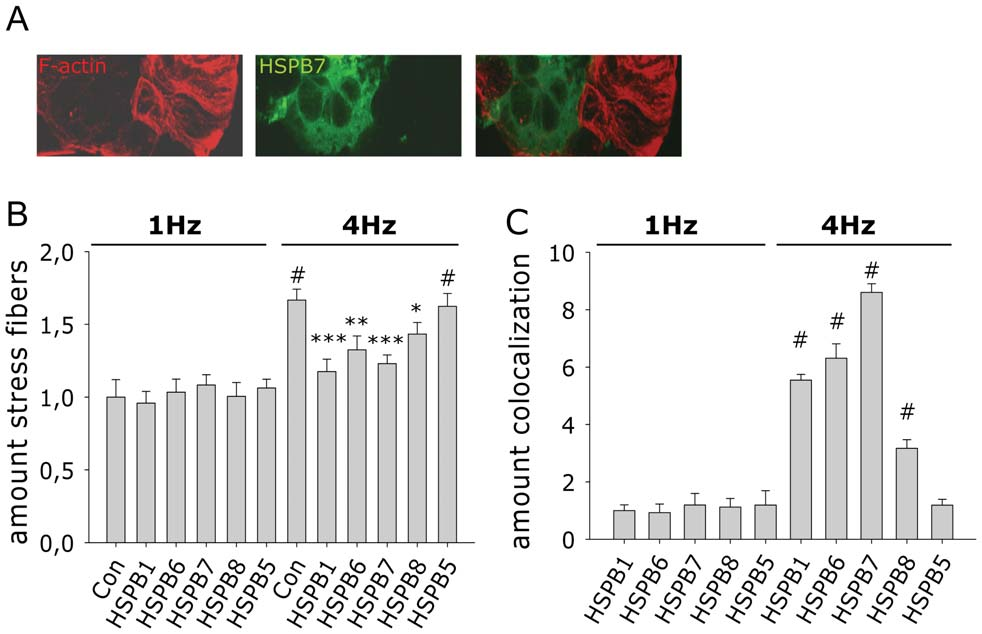
HSPB1, HSPB6, HSPB7 and HSPB8 overexpression is associated with a reduction in the amount of tachypacing-induced F-actin stress fibers in HL-1 myocytes. (A) Immunofluorescent staining of F-actin stress fibers (red) and HSPB7 positive myocytes (green), in tachypaced HL-1 myocytes (4 Hz). HSPB7 positive myocytes reveal less stress fibers. (B) Quantification of the amount of F-actin stress fibers in HSPB transfected HL-1 myocytes after normal pacing (1 Hz) or tachypacing (4 Hz). (C) Quantification of the amount of colocalization of transfected HSPB with F-actin stress fibers. *P<0.05, **P<0.01, ***P<0.001 vs control tachypaced myocytes (4 Hz), #*P*<0.05 vs control normal paced (1 Hz).

Calcium signaling is known to be markedly influenced by the stabilization of the cytoskeleton [13]–[15]. F-actin is one of the major components of the cytoskeleton and located under the plasma membrane to maintain cell shape, rigidity and integrity [16]; [17]. Several HSPB members, including HSPB1, HSPB5, HSPB6, HSPB7 and HSPB8, have been reported to be involved in cytoskeletal stability [18]–[21]. To study if the underlying mechanism for HSPB protection is related to effects on actin, immunofluorescent staining was performed. We observed a 1.7 fold induction in the amount of F-actin stress fibers in tachypaced HL-1 myocytes compared to normal paced control myocytes (Figure 4, 5B), an effect that was significantly reduced by overexpression of HSPB1, HSPB6, HSPB7 or HSPB8 (Figure 5A,B). Overexpression of HSPB5, which did not show protection against tachypacing-induced CaT reductions (Figure 1), also did not lead to a reduction in the amount of tachypacing-induced F-actin stress fibers (Figure 4, 5B). Although in tachypaced HSPB1, HSPB6, HSPB7 or HSPB8 overexpressing myocytes a reduction in the amount of F-actin stress fibers was found, HSPB1, HSPB6, HSPB7 and to a lesser extent HSPB8 colocalized with the F-actin residues after tachypacing and this was not the case for HSPB5 (Figure 4, 5A,C). Taken together, these results suggest that HSPB1, HSPB6, HSPB7 and HSPB8 prevent the formation of F-actin stress fibers in tachypaced HL-1 myocytes, and thereby stabilize the cytoskeleton and myocyte function.

### 3.4. Tachypacing induces RhoA GTPase and ROCK activation, resulting in F-actin formation and reduction in calcium transients

**Figure 6:**
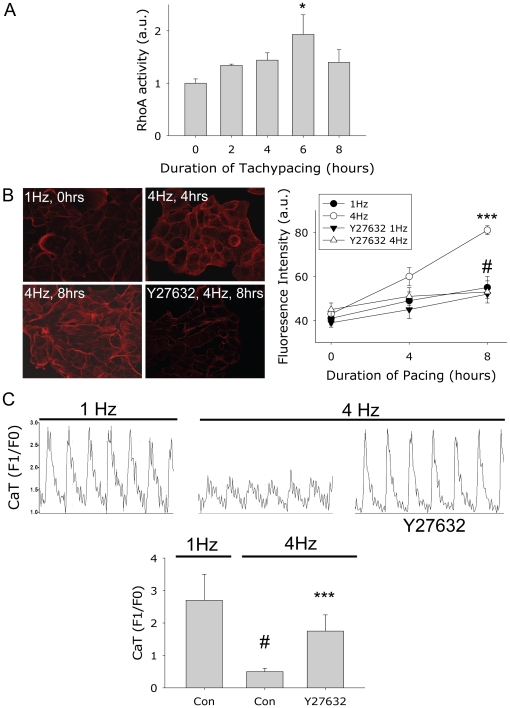
Tachypacing induces gradual activation of RhoA-GTPase and consequently formation of F-actin stress fibers and reduction in CaT in HL-1 myocytes. (A) HL-1 myocytes were tachypaced upto 8 hours and activation of RhoA-GTPase was measured. (B) Left: Examples of immunofluorescent staining of F-actin stress fibers (red) in tachypaced HL-1 myocytes (4 Hz, 4 and 8 hours), normal paced (1 Hz) and tachypaced HL-1 myocytes pretreated with the ROCK inhibitor Y27632 (4 Hz, 8 hours). Right: quantification of the fluorescence intensity of F-actin in the conditions as indicated. (C) Top: Original recordings of CaT in 1 myocyte each from groups indicated. Below: Mean CaT data of normal (1 Hz) and tachypaced (4 Hz) HL-1 myocytes and tachypaced myocytes pretreated with Y27632. ***P<0.001 Y27632 tachypaced vs control tachypaced (4 Hz), #P<0.001 vs control normal paced (1 Hz).

To confirm the role of RhoA GTPase pathway in tachypacing-induced F-actin stress fiber formation and reductions in CaT, HL-1 myocytes were tachypaced for 0-8 hours and RhoA GTPase activity was measured in cell lysates (Figure 6A). A significant induction of the RhoA GTPase activity was observed at 6 hours of tachypacing. In parallel, the amount of F-actin was quantified. A gradual increase in the amount of F-actin was observed during tachypacing, which was prevented by the ROCK inhibitor Y27632 (10 µM) (Figure 6B). In addition, also tachypacing-induced changes in CaT were reduced by Y27632 (Figure 6C), indicating that also this effect is RhoA-mediated.

### 3.5. HSPB8, but not HSPB1, HSPB6, and HSPB7, reduces activation of RhoA GTPases after tachypacing

**Figure 7:**
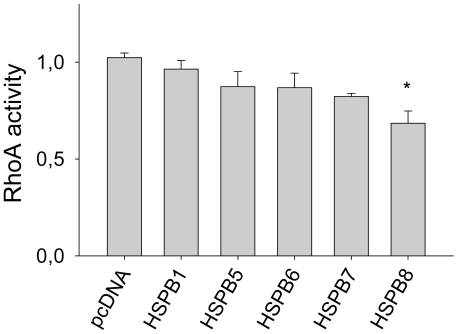
HSPB8 reduces activation of RhoA-GTPase during tachypacing in HL-1 myocytes. HL-1 myocytes were transfected with HSPB1, HSPB5, HSPB6, HSPB7, HSPB8, or empty plasmid (pcDNA) and subjected to tachypacing (4 Hz, 6 hours). Activation of RhoA-GTPase in tachypaced (4 Hz) HL-1 myocytes, transfected with plasmids as indicated, is shown. ^*^ *P*<0.05 vs control tachypaced myocytes (4 Hz).

To test whether the protective effect of HSPB members is related to a direct modulation of the RhoA GTPase activity, RhoA GTPase activity was measured in normal paced (1 Hz) and tachypaced (4 Hz) HL-1 myocytes transfected with the individual HSPB members. None of the HSPB members affected RhoA GTPase activity in 1 Hz paced HL-1 myocytes (Figure S2). Only HSPB8 transfected HL-1 myocytes revealed significantly reduced activation of RhoA GTPase upon 6 hours of tachypacing and all other (protective) HSPB members were ineffective (Figure 7), suggesting that their protective effects against tachycardia remodeling are downstream of RhoA GTPase activation.

### 3.6. HSPB1, HSPB6, and HSPB7 prevent G-to-F actin polymerization

**Figure 8:**
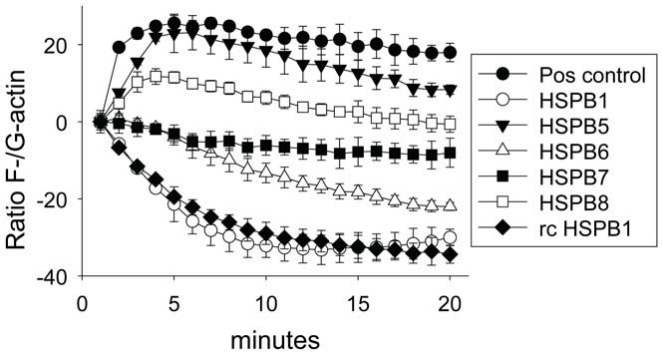
HSPB1, HSPB6, and HSPB7 attentuate F-actin stress fiber formation in vitro. Cell-lysates from HL-1 myocytes transfected with HSPB1, HSPB5, HSPB6, HSPB7, or HSPB8 and recombinant human HSPB1 were used to measure influence of HSPB members on polymerization of G-actin to F-actin and also the depolymerization. ^**^ *P*<0.01 vs lysates of HSPB5 transfected myocytes at 20 minutes incubation, ^***^ *P*<0.001 vs lysates of HSPB5 transfected myocytes at 20 minutes incubation.

To investigate whether HSPB1, HSPB6, and HSPB7, rather than affecting RhoA GTPase activation, may ameliorate the downstream consequences of activated RhoA GTPase, we measured their effect on the polymerization of G-actin to F-actin and also the depolymerization, using an *in vitro* polymerization kit. Base-line fluorescence of G/F-actin ratios were measured for three minutes, after which cell lysates from HL-1 myocytes transfected with the respective HSPB members or recombinant HSPB1 were added (Figure 8). The non-protective HSPB5 was used as a control. When polymerization buffer was added to the baseline G/F-actin, a rapid increase in the conversion of G-to-F actin ratio was observed, indicative of fast actin polymerization. Addition of lysates from HSPB1 transfected cells as well the addition of 0.5 µg recombinant human HSPB1 induced depolymerization of F-actin. Although less effective, lysates from HSPB6 also induce depolymerization, whereas lysates from HSPB7 transfected myocytes prevent actin polymerization but did not show an effect on depolymerization. In contrast to the findings of HSPB1, HSPB6 and HSPB7 in preventing the formation and/or stimulating the depolymerization of F-actin stress fibers, addition of lysates from HSPB8 transfected cells resulted in actin polymerization, although the levels of polymerization were reduced compared to lysates of the non-protective HSPB5 transfected myocytes, which showed near to normal polymerization.

These results together suggest that HSPB1, HSPB6 and HSPB7 may prevent tachycardia remodeling by directly preventing the formation and/or stimulating the depolymerization of F-actin stress fibers downstream of active RhoA GTPase, whilst HSPB8 mainly acts at the level of tachypacing-induced RhoA GTPase activation.

## Discussion

Previously, we showed HSPB1 to protect in HL-1 myocytes against tachycardia remodeling and to preserve normal Ca^2+^ transients as well as the actin cytoskeleton upon tachypacing [2]; [3]. In the current study we found that, in addition to HSPB1, also some other members of the HSPB family (HSPB6, HSPB7 and HSPB8) display protective effects against tachypacing-induced remodeling. Interestingly, all protective HSPB members reduced the formation of F-actin stress fibers, although their modes of action differs. Whereas HSPB8 interfered with tachypacing-induced RhoA GTPase activity, HSPB1, HSPB6, and HSPB7 did not. HSPB1, HSPB6 and HSPB7 were found to directly inhibit G- to F-actin polymerization and/or stimulate depolymerization, indicating a protective role against tachycardia remodeling downstream of RhoA GTPase activation.

### Role of Rho GTPases in induction of AF

The current study revealed a prime role for tachypacing-induced RhoA GTPase activity and consequently F-actin stress fiber formation in reductions in calcium transients. This finding is in line with studies revealing an important role for Rho GTPases, including RhoA and Rac1, in formation of F-actin stress fibers[22] and the initiation of AF [23]; [24]. Consistently, experimental studies showed that activation of RhoA GTPases result in conduction disturbances and cardiac dysfunction similar to those described in AF [25]; [26]. Rho GTPases represent a family of small GTP-binding proteins involved in cell cytoskeleton organization, migration, transcription, and proliferation. Rho GTPases have gained considerable recognition as powerful regulators of actin cytoskeletal organization in the heart [22]. It was observed that active Rho GTPases stimulate the conversion of G-actin to F-actin, which results in cytoskeletal injury including changes in calcium signaling, conduction disturbances and contractile dysfunction [13]–[16], which are all substrates for the development of AF [25]; [26]. Actin stress fiber assembly and contraction are predominantly mediated by Rho-associated serine/threonine kinase (ROCK), a major down-stream effector of the Rho pathway. Consistent with all of the above, we now show that tachypacing activates RhoA and that inhibition of ROCK, its effector of actin polymerization, prevents tachypacing-induced reductions in CaT.

### The HSPB family

Whereas all HSPB members are characterized by the presence of a conserved crystallin domain, this domain is flanked by N- and C-termini that shows large sequence divergence between the members (Table 1) [5]; [8]. Also, the four members (HSPB1, HSPB6, HSPB7 and HSPB8) that we found to have protective effects against tachycardia remodeling show, besides sequence divergence, a number of structural and functional differences (see below). Interestingly, however, all four members, together with the non-protective HSPB5, show high basal expression in heart tissue (Table 1). In addition, three members (HSPB1, HSPB6, HSPB7) seemed to act similar in AF protection, i.e. preventing actin remodeling downstream of RhoA-activation. Only HSPB8 appears to directly affect RhoA-activation. So, the question is what are the characteristics shared by these members and, in addition, what are the differences between them that can explain their protective effects on tachypacing-induced remodeling?

**Table 1:** Characteristics of small HSPB members.

Gene Name	Protein Name	Alternative Name	Sequence Identity	Molecular Size (kDa)	Heat Inducibility	Expression in Heart	Other tissue expression
HSPB1	HSPB1	Hsp25, HSP27, HSP28	100%	22.783	Yes	+++	Uterus, skin, platelets, brain, kidney, some tumor cells
HSPB2	HSPB2	MKBP	36%	20.233	No	+	Skeletal muscle
HSPB3	HSPB3	HSPL27	23%	16.966		+	Skeletal muscle
HSPB4	HSPB4	αA-crystallin, CRYAA, CRYA1	36%	19.909	No	-	Lens of eye, spleen
HSPB5	HSPB5	αB-crystallin, CRYAB, CRYA2	38%	20.159	Yes	++++	Lens of eye, vascular wall cells, lung, kidney, brain, some tumor cells
HSPB6	HSPB6	Hsp20, p20	34%	17.136	No	++	Skeletal muscle, stomach, liver, lung, kidney, platelet
HSPB7	HSPB7	cvHsp	20%	18.611	?	++++++	Skeletal muscle
HSPB8	HSPB8	Hsp22, H11	34%	21.604	Yes	++	Skeletal muscle, stomach, liver, lung, kidney, brain
HSPB9	HSPB9	FLJ27437	19%	17.486	?	-	Testis
HSPB10	HSPB10	ODF1	17%	28.366	?	-	Testis

In cell-free assays, small HSPs have been shown to act as ATP-independent “holdases”, maintaining unfolded or misfolded proteins in a folding competent, non-aggregated state, hereby supporting refolding by ATP-regulated chaperones, in particular the HSP70 machinery [8]. In cellular assays, however, of the four cardioprotective HSPB members, only HSPB1 seems to support such refolding reaction [27]; [28]. Moreover, HSPB5 also shares this activity [28], but did not reveal protective effects against tachycardia remodeling. This finding indicates that such a chaperone-like activity is not of prime importance to the HSPB-mediated protective effects as reported in the current study.

Several members of the HSPB family, including HSPB6, HSPB7 and HSPB8 were recently shown to be able to assist in the clearance of stress-induced misfolded proteins, in part through interaction with (HSPB7) or activation of (HSPB8) the macro-autophagy machinery [28]–[30]. Yet, this activity is not shared by e.g. HSPB1 whilst HSPB9 that also can enhance clearance of misfolded proteins [28], albeit likely via proteasomal degradation [31] had no effect on AF. So, the clearance of misfolded proteins seems not to be a common target of all cardioprotective HSP members.

Another feature shared amongst many HSPB members is their dynamic (de)oligomerisation [5]; [8]. This characteristic has been suggested to be crucial for e.g. the ability of HSPB1 to interact with several cytoskeletal components, including actin, intermediate filaments, and microtubules [7]; [32]. Yet, in cells HSPB7 and HSPB8 do not appear to be present in large oligomeric structures [31]; [33] implying also that this does not edify their protective role against tachycardia remodeling. However, all protective HSPB members can be found in cells as non-oligomeric (most likely dimeric) proteins as well. For HSPB1, dimers have been suggested to be the active species in regulating actin (re)polymerization after stress [34]. Also for HSPB6 stress-induced translocation to actin of the myofibrils has been reported, which has been associated with improved heart function [32]. Also HSPB7 translocates from cytosol to the Z-/I-area of myofibrils, and thereby exerts a protective effect to ischemic stress [21]. This interaction may be mediated via α-filamin, an actin-binding protein [35]. All of this is consistent with our current findings that HSPB1, HSPB6 and HSPB7 are associated with F-actin stress fibers upon tachypacing and the fact that they can directly prevent actin polymerization, an effect that occurs in living cells as a down-stream effect of Rho activation. In addition, the findings also suggest that chaperone-like (refolding or clearance) function and actin protection are distinct, uncoupled functions of these HSPB members. For HSPB8, the remaining AF protecting HSPB member, no direct association with actin and/or microtubules has been reported so far. Although HSPB8 is highly expressed in heart and muscle and anti-HSPB8 antibodies decorate sarcomeres [30], only a weak association with F-actin stress fibers after tachypacing was observed in the current study. Moreover, we only found weak attenuating effects of HSPB8 on actin polymerization. So, the protective effects of HSPB8 against tachycardia remodeling seem distinct from that of the other HSPB members. Consistently, we indeed observed that HSPB8 was the only member that directly affected tachypacing-induced RhoA activation. How HSPB8 may modulate this effect remains an enigma, but maybe its unique role within the HSPB family in activating autophagy [28]; [29] may be important. Autophagy may prevent protein aggregate formation that served as an early trigger for RhoA activation. Indeed, preventing aggregate formation has been suggested as the mode by which HSPB8 can prevents desmin-related cardiomyopathy [36].

The present study demonstrates that RhoA activation plays a central role in tachypacing-induced myocyte remodeling. This remodeling can be prevented by some, but not all, members of the HSPB family. This protection is not directly related to canonical chaperone-like function of these HSPB members, but involves prevention of RhoA activation (HSPB8) or its downstream action on actin remodeling (HSPB1, HSPB6, HSPB7). The findings widen the possibilities for the identification of novel therapeutic approaches directed at RhoA activating components or boosting the expression of one or more of the cardioprotective HSPB members.

## Supporting Information

Figure S1 **Tachypacing induces a progressive reduction in CaT.** A) Original recordings of CaT in 1 myocyte each for a time period as indicated. B) Mean CaT data in myocytes tachypaced at 4 Hz or normal paced at 1 Hz. #P<0.001
(TIF)

Figure S2 **No effect of HSPBs on activation of RhoA-GTPase in normal paced HL-1 myocytes.** HL-1 myocytes were transfected with HSPB1, HSPB5, HSPB6, HSPB7, HSPB8, or empty plasmid (pcDNA) and subjected to normal pacing (1 Hz). Activation of RhoA-GTPase was determined by G-LISA.
(TIF)

Movie S1 **Time-lapse movie shows CaT after 8 hours normal pacing (1 Hz) of HL-1 myocytes.** Images were acquired at 2 ms intervals.
(AVI)

Movie S2 **Time-lapse movie shows CaT after 8 hours tachypacing (4 Hz) of HL-1 myocytes.** Images were acquired at 2 ms intervals.
(AVI)

Movie S3 **Time-lapse movie shows CaT after 8 hours tachypacing (4 Hz) in HSPB1 overexpressing HL-1 myocytes.** Images were acquired at 2 ms intervals.
(AVI)

Movie S4 **Time-lapse movie shows CaT after 8 hours tachypacing (4 Hz) in HSPB2 overexpressing HL-1 myocytes.** Images were acquired at 2 ms intervals.
(AVI)

Movie S5 **Time-lapse movie shows CaT after 8 hours tachypacing (4 Hz) in HSPB3 overexpressing HL-1 myocytes.** Images were acquired at 2 ms intervals.
(AVI)

Movie S6 **Time-lapse movie shows CaT after 8 hours tachypacing (4 Hz) in HSPB4 overexpressing HL-1 myocytes.** Images were acquired at 2 ms intervals.
(AVI)

Movie S7 **Time-lapse movie shows CaT after 8 hours tachypacing (4 Hz) in HSPB5 overexpressing HL-1 myocytes.** Images were acquired at 2 ms intervals.
(AVI)

Movie S8 **Time-lapse movie shows CaT after 8 hours tachypacing (4 Hz) in HSPB6 overexpressing HL-1 myocytes.** Images were acquired at 2 ms intervals.
(AVI)

Movie S9 **Time-lapse movie shows CaT after 8 hours tachypacing (4 Hz) in HSPB7 overexpressing HL-1 myocytes.** Images were acquired at 2 ms intervals.
(AVI)

Movie S10 **Time-lapse movie shows CaT after 8 hours tachypacing (4 Hz) in HSPB8 overexpressing HL-1 myocytes.** Images were acquired at 2 ms intervals.
(AVI)

Movie S11 **Time-lapse movie shows CaT after 8 hours tachypacing (4 Hz) in HSPB9 overexpressing HL-1 myocytes.** Images were acquired at 2 ms intervals.
(AVI)

Movie S12 **Time-lapse movie shows CaT after 8 hours tachypacing (4 Hz) in HSPB10 overexpressing HL-1 myocytes.** Images were acquired at 2 ms intervals.
(AVI)

Movie S13 **Time-lapse movie shows CaT after 8 hrs tachypacing (4 Hz) in mock treated HL-1 myocytes transfected with HSPB6.** Images were acquired at 2 ms intervals.
(AVI)

Movie S14 **Time-lapse movie shows CaT after 8 hrs tachypacing (4 Hz) in siRNA treated HL-1 myocytes transfected with HSPB6.** Images were acquired at 2 ms intervals.
(AVI)

Movie S15 **Time-lapse movie shows CaT after 8 hrs tachypacing (4 Hz) in mock treated HL-1 myocytes transfected with HSPB7.** Images were acquired at 2 ms intervals.
(AVI)

Movie S16 **Time-lapse movie shows CaT after 8 hrs tachypacing (4 Hz) in siRNA treated HL-1 myocytes transfected with HSPB7.** Images were acquired at 2 ms intervals.
(AVI)

Movie S17 **Time-lapse movie shows CaT after 8 hrs tachypacing (4 Hz) in mock treated HL-1 myocytes transfected with HSPB8.** Images were acquired at 2 ms intervals.
(AVI)

Movie S18 **Time-lapse movie shows CaT after 8 hrs tachypacing (4 Hz) in siRNA treated HL-1 myocytes transfected with HSPB8.** Images were acquired at 2 ms intervals.
(AVI)
