# Supplementary figures and images for: HSPB1, HSPB6, HSPB7 and HSPB8 Protect against RhoA GTPase-Induced Remodeling in Tachypaced Atrial Myocytes
Source: PLoS One. 2011 Jun 24;6(6):e20395. doi: 10.1371/journal.pone.0020395 (PMC3123278; doi:10.1371/journal.pone.0020395)

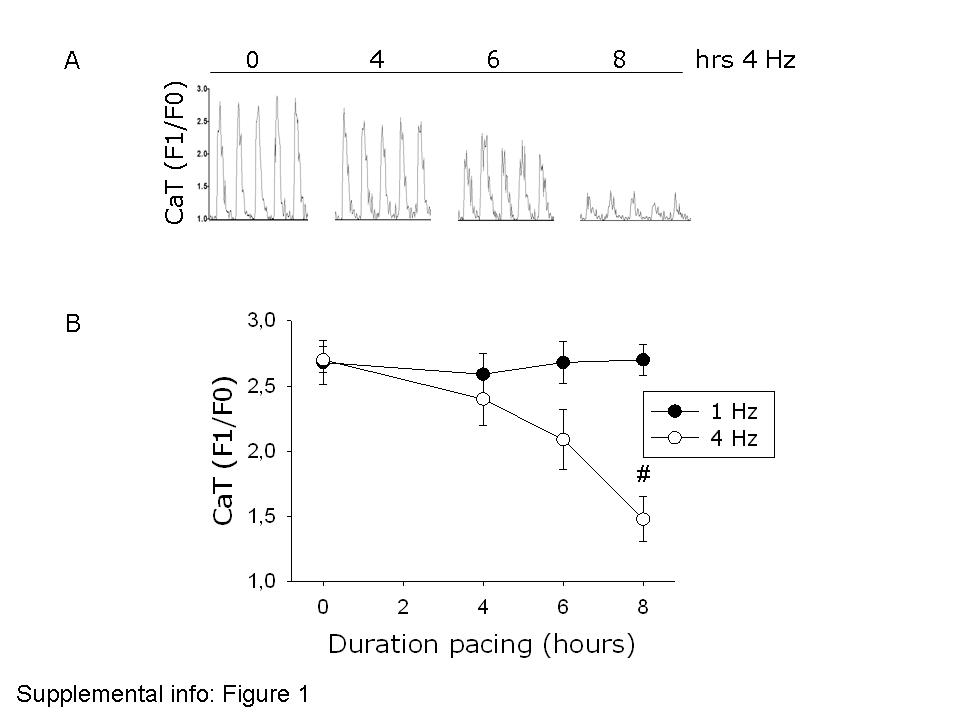

Supplement: Figure S1 — Tachypacing induces a progressive reduction in CaT. A) Original recordings of CaT in 1 myocyte each for a time period as indicated. B) Mean CaT data in myocytes tachypaced at 4 Hz or normal paced at 1 Hz. #P<0.001 (TIF) [file pone.0020395.s001.tif]

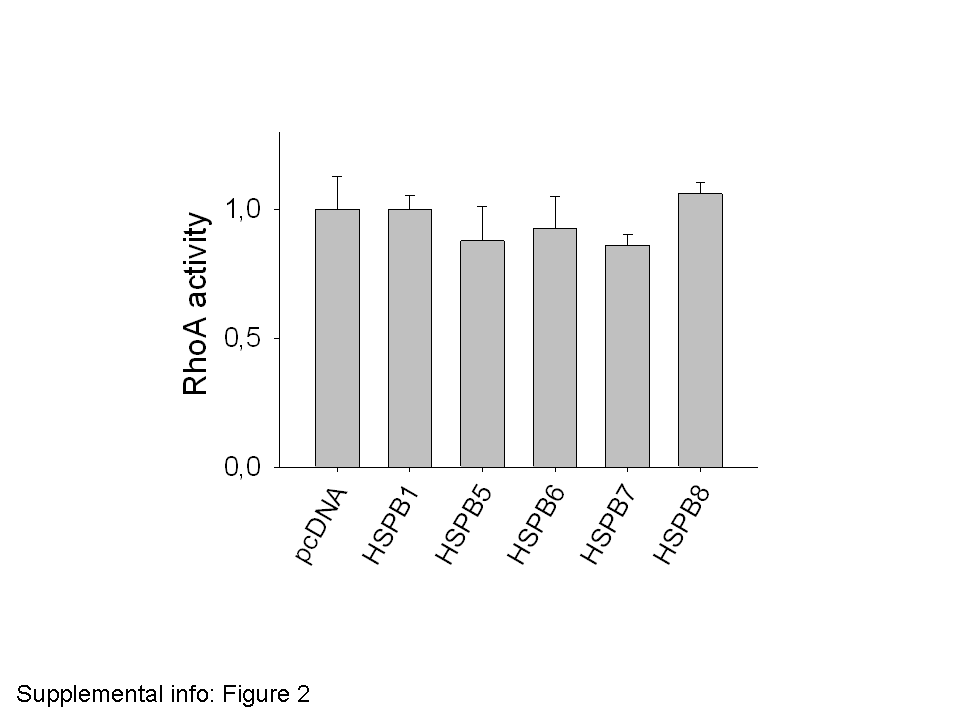

Supplement: Figure S2 — No effect of HSPBs on activation of RhoA-GTPase in normal paced HL-1 myocytes. HL-1 myocytes were transfected with HSPB1, HSPB5, HSPB6, HSPB7, HSPB8, or empty plasmid (pcDNA) and subjected to normal pacing (1 Hz). Activation of RhoA-GTPase was determined by G-LISA. (TIF) [file pone.0020395.s002.tif]
